# MRTF-A can activate Nrf2 to increase the resistance to doxorubicin

**DOI:** 10.18632/oncotarget.14246

**Published:** 2016-12-27

**Authors:** Yao Xu, Ying Luo, Zhen-yu Wang, Xi Li, Peng Zheng, Tong-cun Zhang

**Affiliations:** ^1^ Institute of Biology and Medicine, Wuhan University of Science and Technology, Wuhan, 430065, China; ^2^ Key Laboratory of Industrial Microbiology, Ministry of Education and Tianjin City, College of Biotechnology, Tianjin University of Science and Technology, Tianjin, 300457, China

**Keywords:** MRTF-A, Nrf2, hela, drug resistance

## Abstract

Chemotherapeutic drugs resistance was considered to be the major obstacle for cancer therapy. MRTF-A, co-activators of serum response factor (SRF), promoted tumor cell invasion and metastasis in cancer. So far there has been no relevant reports about MRTF-A’ role in tumor chemotherapy. Here, we reported that MRTF-A overexpression conferred resistance to doxorubicin mediated apoptosis by significantly increasing the expression of Nrf2 which was an important molecule associated with the resistance of anticancer drugs. If MRTF-A was knocked down, could the corresponding results be obtained? Moreover, we showed that overexpression MRTF-A had no remarkable effect to doxorubicin mediated apoptosis in cancer cells when knocking down Nrf2. Further studies showed that MRTF-A regulated the transcriptional activity of Nrf2 by forming a complex with SRF binding to the CarG box which existed on Nrf2 promoter region. On the whole, our study revealed a novel possible resistant pathway to doxorubicin.

## INTRODUCTION

Cervical cancer is one of the most frequent malignant and also the third most common cancer tumors in women with the 5-year survival rate is 69% [1]. Traditional treatment strategies for locally advanced cervical cancer include radical surgery and/or radiotherapy. However, most patients with advanced or metastatic lesions are not amenable to surgery or irradiation and can be treated only by systemic chemotherapy. Chemotherapy, however, is often prematurely discontinued because of drug toxicity and related side effects. Moreover, the prognosis of advanced or bulky tumors remains very poor [2–4]. Doxorubicin, due to its potency against solid tumors, is a commonly used chemotherapeutic agent for the treatment of cervical carcinoma. While drug tolerance is an inevitable problem in recent years despite the application of the combined medication [5]. This article focuses on the possible drug resistance pathway to doxorubicin.

Nrf2, one important factor has an important role in the protection of cell and tissue. Primarily defending cells against metabolic, xenobiotic and oxidative stress, Nrf2 is essential for maintaining tissue integrity. So Nrf2 is regarded as a promising drug target in the chemoprevention of diseases, including cancer. In addition, evidence has accumulated that Nrf2 can increase the tolerance of tumor cells to anticancer drugs [6].

MRTF-A is always thought to be a transcription factor that has a favorable effect on cancer cell migration. While there is little research on the relationship between MRTF-A and drug resistance. In addition, it is well known that MRTF-A regulates the transcription by binding to the CarG box in the promoter of the downstream target gene. Fortunately, we find a CarG box in the promoter of Nrf2. This may suggest that MRTF-A is likely to be able to regulate Nrf2 and thus affect drug resistance. In this paper, we focus on exploring the relationship between MRTF-A and Nrf2, and how the two factors influence drug resistance.

## RESULTS

### MRTF-A could promote the resistance to doxorubicin and the expression of Nrf2

**Figure 1:**
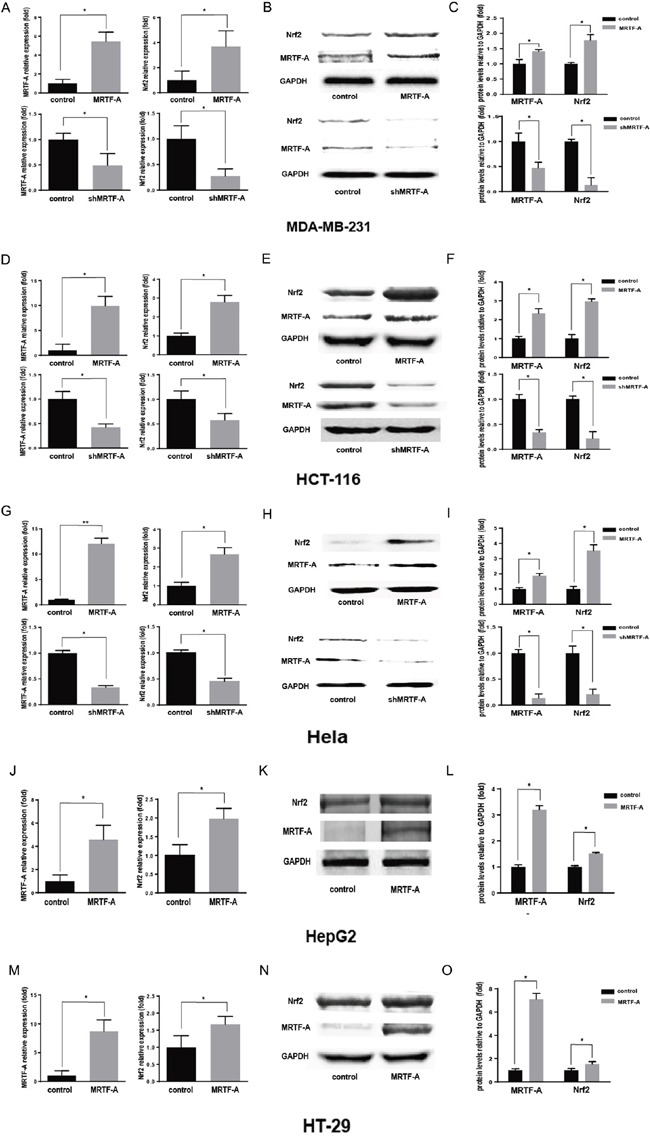
MRTF-A could promote the resistance to doxorubicin and the expression of Nrf2 Realtime RT-PCR was performed to quantitatively measure the mRNA level of MRTF-A and Nrf2. n≥3; *, P < 0.05, **, P< 0.01 vs control cells **A, D, G, J.** and **M.** Western blotting assay for MRTF-A and Nrf2 **B, E, H, K.** and **N.** The figure is representative of at least three independent experiments, and the graphs **C, F, I, L.** and **O.** reveal the densitometric quantification of these experiments (*, P < 0.05).

Five kinds of tumor cells were used to evaluate the levels of endogenous MRTF-A, in which there were three kinds of MRTF-A positive cells and two kinds of negative cells being detected (Supplementary Figure 1A). And the highest level of MRTF-A was expressed in hela cells in this five kinds of tumor cells. To evaluate the relationship between MRTF-A and drug resistance, the plasmids of pcDNA3.1-MRTF-A, no target shRNA and three groups of pLKO.1-shMRTF-A were constructed. The function of these plasmids was tested in hela cells (Supplementary Figure 1B-1C). Because of there was no difference in protein expression in the group of no transfection cells and cells transfected with plasmid skeleton or no target shRNA. The group of no transfection cells could be used as control. And the pLKO.1-shMRTF-A3 was chosen to do the subsequent experiments (Supplementary Figure 1C). In Supplementary Figure 2, a surprising result camed out that the resistance to doxorubicin in five kinds of cancer cells was positively related to the MRTF-A level. To illustrate whether Nrf2 was activated and the relation between Nrf2 and MRTF-A in different tumor cells, we made MRTF-A overexpress (MRTF-A group) and knock down (shMRTF-A group). Within 24h of doxorubicin hydrochloride (Dox) treatment, we used PCR and western blot technology to test the mRNA level of Nrf2 as well as protein. Interestingly, when the expression of MRTF-A raised, we found the level of Nrf2 was significantly increased compared with the control group (Figure 1). However, when knocked down the endogenous MRTF-A, PCR and western blots assay showed the expression of Nrf2 was significantly decreased compared with the control group (Figure 1). These results revealed that MRTF-A can promote the resistance to doxorubicin and the expression of Nrf2 in this five kinds of tumor cells.

### MRTF-A could inhibit the apoptosis induced by doxorubicin

**Figure 2:**
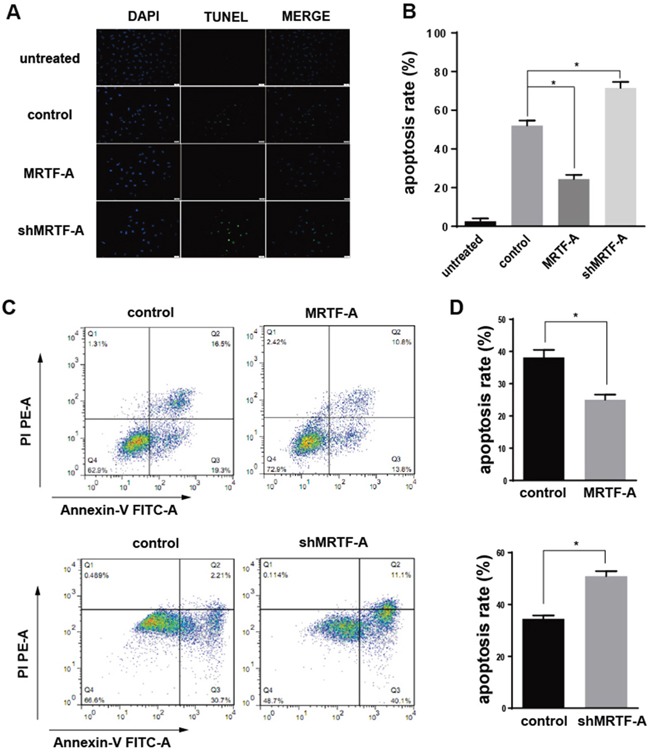
MRTF-A could inhibit the apoptosis induced by doxorubicin **A.** Four different groups of hela cells (untreated group, control group, MRTF-A group and shMRTF-A group) were detected by TUNEL assay treated with 2μM doxorubicin for 24h. The cells in untreated group were normal hela cells without treated with doxorubicin and tansfection. **B.** The apoptosis rates of four different group hela cells were calculated by TUNEL assay. **C.** The effect of MRTF-A on apoptosis of hela cells leading by doxorubicin was determined via Annexin-V/PI double staining assays. The upper right quadrant and lower right quadrant indicated apoptosis cells. **D.** Analysis of percentage of apoptotic cells in the flow cytometry experiment. The apoptosis ratio is presented as a result of early-stage and advanced stage apoptosis. All experiments were repeated at three times, the reprentative images was chosen for presentation (*, p<0.05 compared with control).

It was worth noting that, no matter in the positive cells or the negative cells above showed a same trend in the resistance to doxorubicin as well as the expression of Nrf2 when MRTF-A overexpressed or knocked down (Supplementary Figure 2). So we chose hela cells as a representative to carry out the next phase of the experiments, because it had the highest level of MRTF-A in these five kinds of cells. All of the above tests preliminarily showed that MRTF-A was positively correlated with drug resistance to doxorubicin. In order to further confirm this resistance, TUNEL assay and Flow cytometry assay were used. The TUNEL assay showed a lower apoptosis rate (24.4±2.2%) in the MRTF-A group vs control (52.0±2.7%) after treated with Dox 24h. In addition shMRTF-A group revealed a higher apoptosis rate (71.5±3.2%) vs control (Figure 2A, 2B). At the same time, flow cytometry with FITC-conjugated Annexin V and PI staining was also used to detect the apoptotic cells (Figure 2C). The AnnexinV positive/PI negative cells and Annexin V positive/PI positive cells were considered as early and late apoptotic cells, respectively. Within 24h of 2μM of Dox treatment, the total percentage of early and late apoptotic cells were 25±0.9452% in case of hela cells treated with pcDNA3.1-MRTF-A. And the control was 38.17±1.357%. When knocking down the expression of MRTF-A, the total percentage of early and late apoptotic cells were 50.87±1.167%. While the apoptosis rate in the control was 34.40±0.7906%. Both of TUNEL assay and Flow cytometry assay showed that the percentage of apoptotic cells induced by Dox treatment was significantly increased in the shMRTF-A group compared with control group. While, the MRTF-A group did the opposite, the number of apoptotic cells was decreased. These results indicated that MRTF-A was resistant to the Dox-induced apoptosis of hela cell. All the groups were treated with 2μM doxorubicin.

### MRTF-A activated the expression of Nrf2 target genes to prevented apoptosis

**Figure 3:**
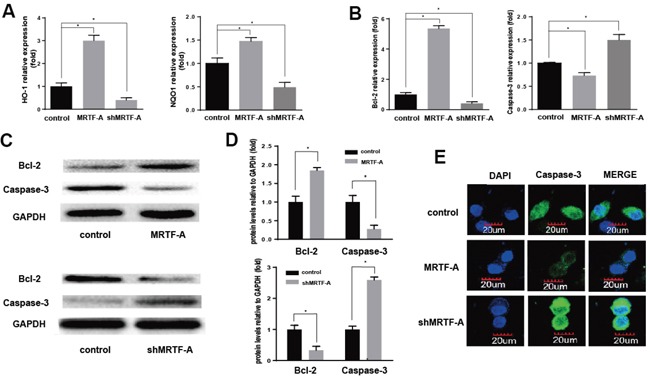
MRTF-A activated the expression of Nrf2 target genes to prevented apoptosis **A, B.** HO-1, NQO-1, Bcl-2 and Caspase-3 expression levels (relative to that of GAPDH) were individually determined by real-time RT-PCR. n≥3; *, P < 0.05 vs control cells; Verification of Bcl-2 and Caspase-3 expression by Western blot **C, D.** The significantly change of HO-1, NQO-1, Bcl-2 and Caspase-3 protein levels vs GAPDH. n≥3; *, P < 0.05 vs control cells; The expression and location of caspase-3 was detected by cellular immunofluorescence **E.** All the control cells were no transfected cells.

Nrf2 and some of its downstream target genes formed a signaling pathway regulating drug resistance. Functional activation or inhibition of this signaling pathway with/without MRTF-A in response to doxorubicin was evident and showed in Figure 3. As shown in Figure 3A, the mRNA expression of HO-1 and NQO1 was up-regulated significantly in the MRTF-A group compared with the control group. However, the shMRTF-A group showed the opposite result. In contrast to the control group, the mRNA level of HO-1 and NQO1 were decreased. Then we investigated Bcl-2, Caspase-3 mRNA and protein levels by PCR and western blot analysis. As shown in Figure 3B-3D, Bcl-2 decreased significantly in the MRTF-A group compared to those in the control group. But the result of caspase-3 shows opposite. To further confirmed anti-apoptotic effects of MRTF-A, we conducted an immunofluorescence analysis for caspase-3. The results of MRTF-A group and shMRTF-A group compared to the control were consistent with the previous (Figure 3E).

### MRTF-A affected resistance to the doxorubicin by adjusting the Nrf2

**Figure 4:**
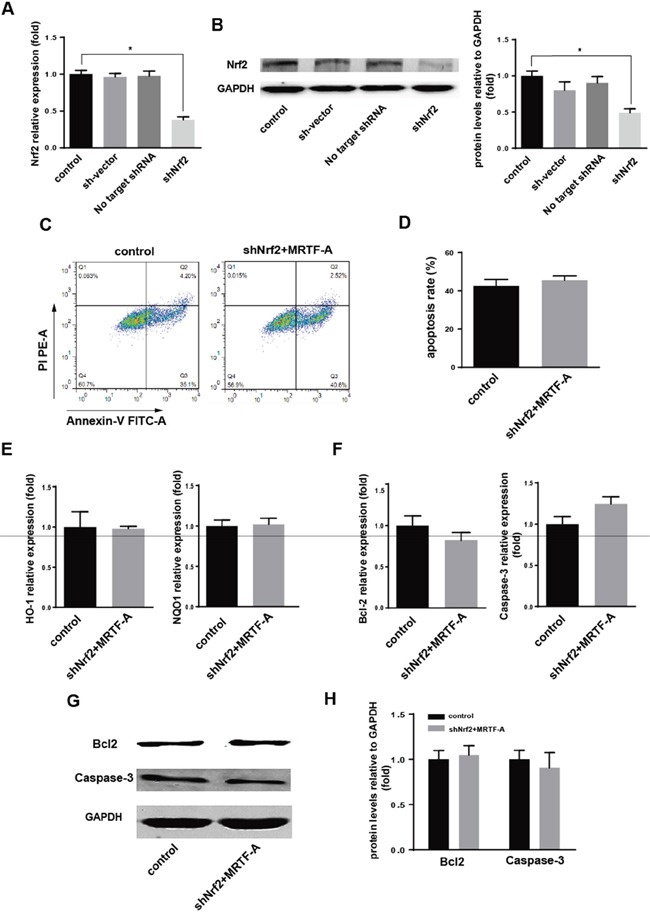
MRTF-A affected resistance of hela to the doxorubicin by adjusting the Nrf2 **A, B.** The effection of shNrf2 was identified by real-time RT-PCR and Western blot. n≥3; *, P < 0.05 vs control cells; The speculation whether the role of MRTF-A was achieved through the Nrf2 was proved by Flow cytometry **C, D.** real-time RT-PCR **E, F.** and Western blot **G, H. (C)** Flow cytometry to detect the number of apoptotic cells; (D) was a quantitative analysis to the result of flow cytometry. n≥3; The levels of these markers downstream were detected by real-time RT-PCR (E, F) and Western blot (G, H). n≥3. All the control cells were no transfected cells.

The above results confirmed that MRTF-A could activate Nrf2 and its downstream genes. Whether it was true that MRTF-A affected resistance through the regulation of Nrf2? The function of no target shRNA and shNrf2 plasmids were tested in hela cells (Figure 4A-4B). Because of there was no difference in mRNA and protein expression in the group of no transfection cells and cells transfected with plasmid skeleton or no target shRNA. The group of no transfection cells could be used as control. Firstly 2μg shNrf2 was tansfected to hela cells (shNrf2 group). Then we tansfected 2μg MRTF-A to the above hela cells and the normal hela cells as control. Flow cytometry with FITC-conjugated Annexin V and PI staining was used to detect the apoptotic cells (Figure 4C). Annexin-V/PI double staining assays showed the percentage of apoptotic cells induced by doxorubicin treatment had no significant difference between the two groups (39.0±1.435% vs 43.12±0.8206%) (Figure 4D). To further prove our conjecture, we used PCR technology to test mRNA levels’ change of HO-1, NQO1, Bcl-2 and Caspase-3. At the same time, western blot was also used to detect the protein level of Bcl-2 and Caspase-3. The results confirmed that, when the Nrf2 was knocked down, MRTF-A can't regulate the drug resistance in doxorubicin-treated hela cells (Figure 4E-4H). This is consistent with results of previous Flow cytometry.

### MRTF-A effected the Nrf2 expression through binding to the CarG box of Nrf2 promoter

**Figure 5:**
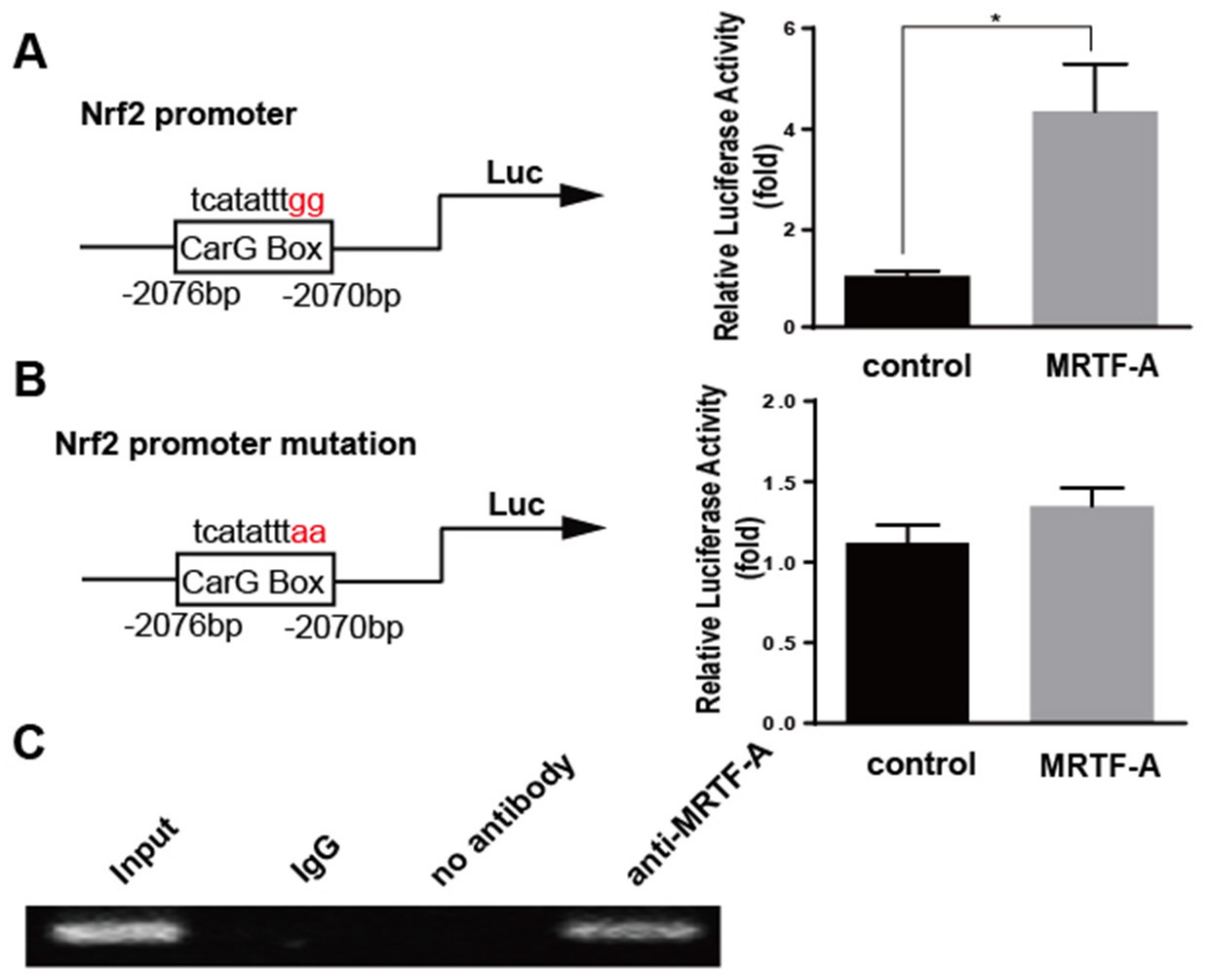
MRTF-A effected the Nrf2 expression through binding to the CarG box of Nrf2 promoter The promoter region of Nrf2 was inserted into pGL-3(Nrf2-luc), and point mutation of Nrf2 promoter region was constructed by circular PCR(mut-Nrf2-luc). For transfection experiments, the Cos-7 cells were transfected with 0.8 μg MRTF-A and 0.2 μg Nrf2-luc or mut-Nrf2-luc. Luciferase assay was performed (*, P<0.05, n=3) **A.** and **B.** The vector pcDNA3.1 alone was used as a negative control. C. Chromatin immunoprecipitation (ChIP) assay was used to determine whether MRTF-A can change Nrf2 transcriptional activity through binding on CarG box.

Previous reports had confirmed that MRTF-A played a regulatory role on downstream target genes through binding to the CarG box. Exactly there was one potential CarG box on Nrf2 promoter region. Then the effect of MRTF-A on the transcriptional activity of Nrf2 was analyzed by luciferase assay. The results prompted that the transcriptional activity of Nrf2 promoter could be up-regulated by MRTF-A (Figure 5A). However, the Nrf2 transcriptional activity was not affected with a mutated CarG box (Figure 5B). The results might indicate MRTF-A affected the Nrf2 transcriptional activity through binding to the CarG box. To further tested the mechanism of this regulation, we used chromatin immunoprecipitation (ChIP) to investigate in normal hela cells. Datas showed that MRTF-A could be combined with CarG box, consistent with results of luciferase assay (Figure 5C).

## DISCUSSION

Chemotherapy was one of the important means for the treatment of cancer. And resistance was a major obstacle hindering the effect of chemotherapy, which related to many kinds of cancer drugs used commonly in the clinical. In recent years, the research progress in the mechanism of multidrug resistance of cancer was more concentrated in the aspects of drug transport pump and cell apoptosis. However more important information should be gained through the study of cancer drug resistance mechanisms to help patients overcome drug resistance and improve the effectiveness of cancer chemotherapy, while helping to find more effective anti-cancer target molecules.

A large number of studies showed that Nrf2 was an important molecule associated with the drug resistance of anticancer drugs. In different cancer cells, the mutation of Nrf2 and/or Nrf2-associated inhibitors Keap1 made the increase of Nrf2 [7–10]. The mechanism of drug resistance of Nrf2 was as follows: Nrf2 as a nuclear transcription factor transported into the nucleus and binding to the AREs sequence, activating the molecules downstream, such as antioxidant molecules, detoxification protein and inhibiting cell apoptosis (Bcl-2) and multidrug resistance drug associated protein (MRP), and then participated in the oxidative stress reaction to make cancer cells resistant to drugs [11–13]. Nrf2 and drug resistance related studies had been developed rapidly, but we knew little about the relationship between MRTF-A and drug resistance. MRTF-A was generally considered to have a wide range of effects on cell growth, migration, differentiation, and apoptosis [14–15]. It could form a complex with SRF protein then regulated the transcription of downstream target genes by binding to highly conserved cis regulatory element CC (AT) _6_GG (CarG box) in the promoters of target genes [17–19]. According to this, we analyzed the sequence of Nrf2 promoter. Luckily, we found a CarG box. Then we performed MRTF-A over expression and silence in hela cells, to observe the level of endogenous Nrf2. It was exciting to find that the expression of Nrf2 was positively correlated with the level of MRTF-A. At this point we had reason to believe that MRTF-A could affect the drug resistance. And the following experiments confirmed our speculation.

We found that the sensitivity of tumor cells with MRTF-A over expression to doxorubicin was significantly lower than that of normal tumor cells, and the up-regulation of anti-apoptotic genes and genes about drug resistance were also observed. However, when MRTF-A was disturbed, the above tendency was the opposite. All these showed that MRTF-A could increase the resistance of tumor cells. While this effection of MRTF-A was certainly achieved through regulating Nrf2? Whether there also existed other factors downstream of MRTF-A enhancing this resistance? So we conducted a new experiment. We firstly silenced Nrf2 and then transfected MRTF-A, observing the sensitivity of tumor cells to doxorubicin. It was interesting that we found MRTF-A induced drug resistance disappeared. This showed that the drug tolerance caused by MRTF-A was directly achieved through the regulation of Nrf2. To address how MRTF-A regulated Nrf2 transcription, we constructed Nrf2 promoter luciferase reporter plasmid, which contained CarG box, and investigated the effect of MRTF-A on Nrf2 transcription. MRTF-A promoted target genes transcription driven by serum response factor (SRF), which recognizes CarG box DNA sequence in target genes promoter. The results showed that MRTF-A enhanced transcription of Nrf2. But when CarG box was mutated, the effect was gone. This meant that MRTF-A regulated the transcriptional activity of Nrf2 by complex with SRF binding to the CarG box. Chip assay also confirmed the above statement.

In summary, our study provided some evidence that MRTF-A could significantly increase the tolerance of tumor cells to doxorubicin. And this effect was achieved by adjusting Nrf2. Loss of function of Nrf2 strongly inhibited MRTF-A increasing the tolerance to doxorubicin. Furthermore, reporter assays with Nrf2 promoter indicated that MRTF-A could synergistically activate Nrf2 transcription and enhance the mRNA level of Nrf2. In addition, chip assay demonstrated MRTF-A promoted the transactivity of Nrf2 by the formation of the MRTF-A/SRF/CarG complex. These findings revealed a new possible transcription factors signaling pathway of the tolerance to doxorubicin. We hoped this work could be useful to the future researches about tumor drug resistance.

## MATERIALS AND METHODS

### Cell culture

All tumor cells used in this study were obtained from America Type Culture Collection. MDA-MB-231 cells (ATCC, Number HTB-26™), HepG2 (ATCC, Number HB-8065™) and Hela (ATCC, Number PTA-5659) were seeded in Dulbecco's modified Eagle's medium-high glucose (DMEM-HG, Hyclone Co.) supplemented with 10% fetal bovine serum (FBS, GIBCO) at 37°C in humified air with 5% CO2. HCT-116 (ATCC, Number CCL-247™) and HT-29 (ATCC, Number HTB-38™) were seeded in McCoy's 5a Medium (Boster) supplemented with 10% fetal bovine serum (FBS, GIBCO) at 37°C in humified air with 5% CO2. Cos-7 cells (ATCC, Number CRL-1651) were cultured in Dulbecco's modified Eagle's medium-low glucose (DMEM-LG, Hyclone Co.) containing 10% FBS (GIBCO).

### Cell transfection

For transfection experiments, the cancer cells were cultured in growth medium without antibiotics at 60% confluence for 2 days, and then transfected with transfection reagent (FuGENE® HD, Roche) according to manufacturer's instructions. After incubation for 6h, the medium was removed and replaced with normal culture medium for 24h. For immunocytochemistry assay, cancer cells were cultured in 24-well plate and 2μg DNA was added to each well of a 24-well plate. For qRT-PCR assay, cancer cells were cultured in 6-well plate and 4μg DNA was needed in each well. For western blotting, cancer cells were cultured in 6-well plate and 4μg DNA is needed in each well. Then, the immunocytochemistry assay, PCR, luciferase analysis and western blotting were performed as follows described.

### Reverse-transcription polymerase chain reaction (RT-PCR) and quantitative real-time RT-PCR (qRT-PCR)

Total RNA was isolated from cells using Trizol reagent (Invitrogen, Carlsbad/CA), and two microgram of the sample were reverse-transcribed using M-MLV reverse transcriptase (Promega, Madison/WI). Glyceral-dehyde-3-phosphate dehydrogenase (GAPDH) was used as an internal control to show equal loading of the cDNA samples. Real-time PCR was performed in an Applied Biosystems Step One™ Real-Time PCR System. Fast SYBR® Green Master Mix was obtained from Applied Biosystems. Data were shown as relative expression level after being normalized by GAPDH. The primers for the PCR analysis are listed in Table 1.

**Table 1:** Real-time PCR primer sequences

Genes	Primers for qRT-RCR
GAPDH	F:TCAAGAAGGTGGTGAAGCAG
	R:AGGTGGAGGAGTGGGTGTCG
Nrf2	F:AGTCAGCGACGGAAAGAGTA
	R:CTGGGAGTAGTTGGCAGATC
MRTF-A	F:AAGGAACCACCTGGCTATGA
	R:CTCCGCTCTGAATGAGAATGT
HO-1	F:TGACACCAAGGACCAGAGC
	R:CATCGGAGAAGCGGAGC
NQO1	F:GAAGAAACGCCTGGAGAATA
	R:CTGGTTGTCAGTTGGGATGG
Bcl-2	F: GCCTTCTTTGAGTTCG
	R: CCCAGCCTCCGTTAT
Caspase-3	F: TGGTTCATCCAGTCGCTTTG
	R: ATTCTGTTGCCACCTTTCG

### Protein extraction and western blotting

Proteins prepared were separated by SDS-PAGE and transferred to nitrocellulose membranes. The membranes were immunoblotted with rabbit anti-MRTF-A(Santa cruz), rabbit anti-Nrf2(Santa cruz), mouse anti-Bcl-2(Santa cruz), mouse anti-caspase-3(Santa cruz) antibodies overnight at 4°C, and then incubated with IRDyeTM-800 conjugated anti-mouse and anti-rabbit secondary antibodies (Jackson ImmunoResearch Laboratories) for 30 min at RT. The specific proteins were visualized by Odyssey Infrared Imaging System (Gene Company Limited). GAPDH expression was used as an internal control to show equal loading of the protein samples.

### MTT assay

Cell growth was estimated by a modified MTT assay. As a measurement of cell growth, the cells were seeded onto 96 well dish and grown in medium containing10% FBS. After the cells were treated daily with Dox (0.1, 0.25, 0.5, 1, 2, 4, 8, 16, 32μM) for 24 h, the MTT reagent (2.5 mg/mL) was added and the optical density (570 nM) was measured by ELISA reader.

### Detection of DNA fragmentation by TUNEL method

DNA fragmentation was detected by the DeadEnd™ Fluorometric TUNEL System (Promega) as described previously [19]. The TUNEL reaction was performed according to the manufacturer's instructions. For negative controls, TdT enzyme was not included in the incubation buffer. The apoptotic index was quantified by calculating the number of positive TUNEL cells from 1000 cells in five different microscopic fields.

### Annexin V assay of cell apoptosis

Cells were cultured in six-well plates and were treated with the indicated compounds, trypsinized and collected. The collected cells were washed with PBS, resuspended in binding buffer, and stained with Annexin V-PE (FITC) and 7-AAD (PI) for 15 minutes according to the manufacturer's protocol from Becton Dickinson. Fluorescence was estimated with a Becton Dickinson flow cytometer.

### Immunocytochemistry assay

The cells after treatment were fixed in 4% paraformaldehyde for 15 min and then blocked with normal goat serum for 20 min at room temperature (RT). After incubation with primary antibodies ( mouse anti-caspase-3, Santa Cruz) in a humid chamber overnight, cells were incubated with appropriate secondary antibodies fluorescein isothiocyanate (FITC)-conjugated goat anti-mouse IgG for 30 min at 37°C. mouse anti-caspase-3 IgG diluted 1:200 in phosphate-buffered saline (PBS), whereas FITC-conjugated goat anti-mouse IgG diluted 1:100 in PBS. After washing with PBS, the samples were observed under laser scanning confocal microscope (OLYMPUS, Japan).

### Luciferase constructs, site-mutation, and luciferase assay

Nrf2-luc: Nrf2 5'-flanking region (−2196/0) was fused to pGL-3 luciferase coding sequence. Mut-Nrf2-luc: equal to pGL-3-Nrf2, except that MRTF-A binding CarG box site was mutated from TCATATTTGG to TCATATTTAA. The cells (2 × 10^5^/well) were plated in twenty -four-well plates. Cos-7 cells were cotransfected with MRTF-A expression plasmids (pcDNA3.1-MRTF-A) or control vector (pcDNA3.1) in combination with Nrf2-luc, or mut-Nrf2-luc. Cells were harvested 24 h after transfection and luciferase activity was measured using the Dual luciferase Assay System (Promega, Madison/WI). Results were expressed as a fold induction relative to the cells transfected with the control vector (pcDNA3.1) after normalization to renilla activity. In the result of the dual luciferaseassay, all columns represent the mean result of three independent experiments and the error bars represent the standard deviation.

### Chromatin immunoprecipitation (ChIP) assay

We used a commercial chip assay kit (Upstate Biotechnology, Waltham, USA) by following the manufacturer's instructions. After the treatment, each test team incubated with 1% formaldehyde to cross-link DNA–protein complexes. After washing with ice-cold PBS for three times, cells were lysed in SDS lysis buffer. Then lysate was sonicated to shear DNA to 200~1000 bp fragments. Then we use an anti-MRTF-A antibody to immunoprecipitate the cross-linked protein at 4 °C overnight. IgG acted as the negative control. The DNA was used as a template for PCR and utilized the MRTF-A binding sites. The PCR products were separated on 1% agarose gel. The PCR primer sequences was as follow : forward5′- GGAGCCTGTAAATCTGT -3′, reverse 5′-GACTGCATTCTGGACTAT -3′.

### Statistical analysis

Data were expressed as mean ± SD, accompanied by the number of experiments performed independently, and analyzed by t-test. Differences at P<0.05 were considered statistically significant.

## SUPPLEMENTARY MATERIALS FIGURES AND TABLES


