# Supplementary material for: MRTF-A can activate Nrf2 to increase the resistance to doxorubicin
Source: Oncotarget. 2016 Dec 27;8(5):8436–46. doi: 10.18632/oncotarget.14246 (PMC5352412; doi:10.18632/oncotarget.14246)
Supplement: Supplementary file 1 [file oncotarget-08-8436-s001.pdf]

MRTF-A can activate Nrf2 to increase the resistance to doxorubicin

SUPPLEMENTARY FIGURES

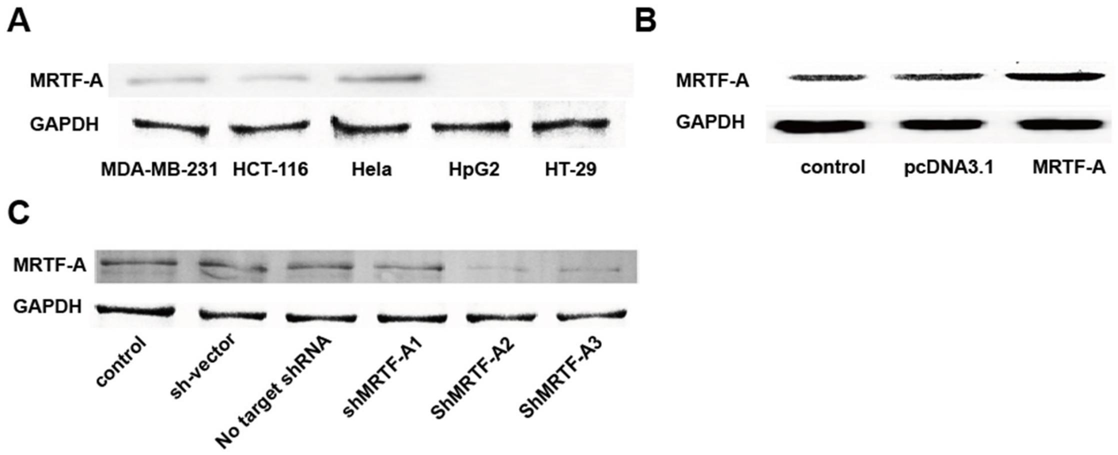

Supplementary Figure 1: A. Expression of endogenous MRTF-A in different tumor cell lines. Western blot was used to detect the protein level of MRTF-A in MDA-MB-231, HCT-116, Hela, HepG2 and HT-29. **B.** Protein levels'change of MRTF-A dectected by western blot after transfected with pcDNA3.1 and pcDNA3.1-MRTF-A in hela cells. Untansfected cells as control; **C.** Detection of the effect of interference plasmid. Protein levels'changes of MRTF-A dectected by western blot. Hela cells transfected with pLKO.1 were named as sh-vector group. The shMRTF-A1, shMRTF-A2 and shMRFT-A3 groups were tansfected with three different kinds of MRTF-A interfering plasmids. And the no target shRNA was constructed by inserting a non-targeting sequence into pLKO.1. Notransfected cells as control.

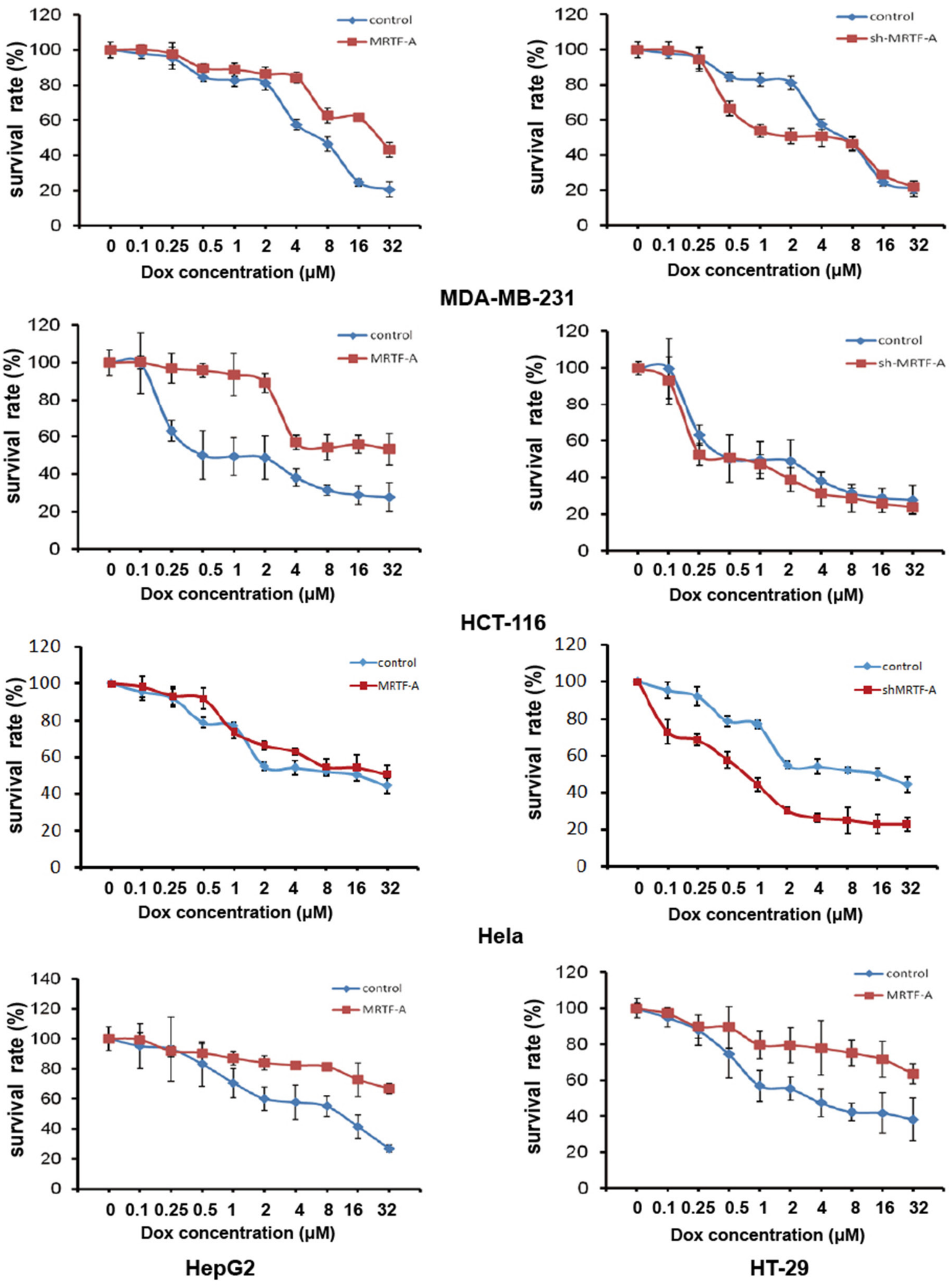

Supplementary Figure 2: Detection of the drug resistance of five kinds of tumor cells. Five kinds of tumor cells were treated with different concentrations (0.1, 0.25, 0.5, 1, 2, 4, 8, 16, 32μM) of doxorubicin for 24h. Cell surviving rate was determined using MTT assay. Data (mean ± SD) represent the mean value of three independent experiments.
